# Valorization of Cactus Cladodes (*Opuntia ficus indica*): The Effects of Different Drying Methods on Antioxidant Compounds and Physicochemical Characteristics

**DOI:** 10.1002/fsn3.72461

**Published:** 2026-11-03

**Authors:** Şehriban Gül Bağirsakci, Erkan Karacabey, Salih Karasu

**Affiliations:** ^1^ Department of Gastronomy and Culinary Arts, Faculty of Art and Design Antalya Belek University Antalya Türkiye; ^2^ Department of Food Engineering, Faculty of Engineering and Natural Sciences Suleyman Demirel University Isparta Türkiye; ^3^ Department of Food Engineering, Faculty of Chemistry and Metallurgy Yildiz Technical University Istanbul Türkiye

**Keywords:** antioxidant capacity, ascorbic acid, drying, flavonoid, flavonol, phenolic profile

## Abstract

*Opuntia ficus indica* is among the most widely cultivated and consumed cactus species worldwide. Cactus cladodes were dried using convectional drying, microwave drying, and ultrasound pretreatment combined with vacuum‐assisted convectional drying. Total phenolic, flavonoid, and flavonol contents; phenolic profile; ascorbic acid content; antioxidant capacity; color, texture were analyzed in fresh and dried samples, and microstructural changes were examined by SEM. Drying process is effective on phenolic compounds (*p* < 0.05). Ultrasound pretreatment has been important for total phenolic compound preservation. Among the dried samples, the highest total phenolic content was observed in ultrasound pretreated vacuum‐assisted convectional drying with 70°C. Drying treatments significantly affected the Δ*E* values (*p* < 0.05), with the highest color difference from the fresh sample observed in the ultrasound pretreated vacuum‐assisted convectional drying treatment at 80°C (23.62). Significant decreases were observed in the flavonoid and vitamin C amounts of the samples in the ultrasound pretreated drying method in pure water. As a result of the phenolic profile analysis, a total of nine components were determined. The antioxidant capacity of bioactive compounds did not show a statistically significant change according to different drying processes and drying parameters. In addition, flavonol amounts with drying processes were not affected by drying processes. The change in the skin strength value was found to be statistically insignificant in convectional drying and microwave drying applications. In the SEM analysis, the effects of different drying techniques and the parameters used in drying techniques on the product texture were revealed.

## Graphical abstract

*Opuntia ficus indica* cladodes were sliced to 5 mm and dried using conventional, microwave, and ultrasound pretreated vacuum‐assisted convectional drying. Drying significantly affected total phenolic content (*p* < 0.05), with the highest value obtained using ultrasound pretreated vacuum‐assisted convectional drying at 70°C. Nine phenolic compounds were identified by HPLC. Ultrasound pretreatment enhanced phenolic preservation but decreased flavonoid and vitamin C contents and affected color, texture, and microstructure. Antioxidant capacity was not significantly affected by drying. Conventional and microwave drying, with shorter drying times and comparable antioxidant capacity, may provide alternative approaches for cactus cladode processing.

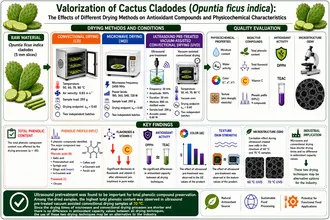

## 1 Introduction

*Opuntieae* is an endemic plant of the Americas (López et al. 2009). In addition, it grows in Mediterranean countries such as Turkey, Italy, Egypt, and Spain; in Middle East countries such as Israel and Jordan; in Central and South Africa; as well as in India and Australia (Stintzing and Carle 2005).

While many cactus species are not consumed, *Opuntieae* species can be consumed (FAO 2017, 2021). The cactus plant is used as a source of fruit and vegetables, fodder for animals, and functional food for various health problems (El‐Mostafa et al. 2014).

*Opuntia ficus indica* (*O. ficus indica*) is one of the most produced, distributed, and consumed cactus species. It grows wild in the Mediterranean and Aegean regions of Turkey, but it does not have enough potential in terms of consumption and processing into different products (Belviranlı et al. 2019).

In recent years, the trend toward natural products such as fruits, vegetables, herbs, and oilseeds has increased as sources of nutraceutical antioxidants and functional products. In this context, the fruit of the *O. ficus indica* plant (prickly pear) and the leaves (cladodes) of the plant known as “nopales” or “nopal” are used as food (Ayadi et al. 2009).

*O. ficus indica* fresh cladodes are preferred by people due to their low cost, delicious sour taste, and nutritional and functional properties. In this context, fresh cladodes of *O. ficus indica* are considered a vegetable with nutritional and medicinal properties (Bakewell‐Stone 2023).

It has been determined to have antidiabetic and anti‐hyperlipidemia effects and is an adjunct therapy for obesity. Considering these nutraceutical benefits and rich content in functional substances, cactus leaves attract the attention of researchers from various parts of the world. FAO declared that “it is time to put cactus on food menus and it will be one of the leading plants for the 21st century” (Stintzing and Carle 2005; FAO 2017).

Although cactus cladodes are available year‐round, postharvest losses can exceed 50% depending on varying storage conditions, posing a significant challenge (Feugang et al. 2006; dos Santos et al. 2024). Food preservation plays a critical role in ensuring food security, reducing postharvest losses, and extending the shelf life of agricultural products. Among preservation techniques, drying is one of the oldest and most widely applied methods, as it suppresses microbial growth and enzymatic activities by reducing moisture content, thereby improving product stability and facilitating storage, transportation, and trade (Pathare and Jayasuriya 2026).

The aim of this study was to improve the quality attributes of *O. ficus indica* cladodes, an underutilized plant resource naturally distributed across Mediterranean regions, including Turkey, as well as the Middle East and Southern Africa, through the application of different drying techniques and processing conditions. Convectional drying, microwave drying, and ultrasound‐pretreated vacuum‐assisted convectional drying were selected because they represent widely used drying technologies with distinct heat and mass transfer mechanisms, processing efficiencies, and impacts on product quality. Convectional drying is commonly applied in the food industry due to its simplicity and cost‐effectiveness, whereas microwave drying offers rapid volumetric heating and reduced processing times. Vacuum‐assisted convectional drying enables moisture removal at lower temperatures, potentially minimizing thermal degradation of heat‐sensitive compounds. These methods were comparatively evaluated in terms of their effects on color and textural properties, total phenolic content, and antioxidant capacity. Furthermore, the influence of ultrasound pretreated vacuum‐assisted convectional drying on the retention of bioactive compounds and overall product quality was investigated. Ultrasound treatment was included because of its potential to enhance mass transfer through microstructural modifications, thereby improving drying efficiency and the preservation of quality attributes (Turan et al. 2023). The results contribute to the limited knowledge regarding the processing of cactus cladodes and provide a scientific basis for their utilization as functional food ingredients and value‐added products. In addition, the findings offer practical guidance for the optimization of drying processes and support the potential industrial application of cactus cladodes in food and agro‐industrial sectors.

## 2 Materials and Methods

### 2.1 Plant Material

Cactus cladodes (*O. ficus indica*) growing wild in the same field in Antalya/Turkey were collected in May during the morning hours (08:00–10:00 a.m.). Medium‐mature cactus cladodes (18–22 cm) were identified using a ruler and selected for analysis. Cactus leaves were washed, cleaned, and separated from their spines. Cladodes were sliced to a 5 mm slice thickness with a slicing machine (HBS‐200; Arisco, Turkey) (Rodriguez‐Felix and Cantwell 1988; Contreras‐Padilla et al. 2012; Özçelik and Karacabey 2019).

### 2.2 Convectional Drying (CD)

Convectional drying was performed using a tray‐type drying oven (MSD2.50.D; Microtest, Turkey) equipped with temperature control and a forced‐air circulation system. The airflow rate was maintained constant throughout the drying process. Air velocity inside the drying chamber was measured at 10 different locations, and the average airflow velocity was determined as 0.83 m s^−1^.

Cactus cladode slices (5 mm thickness) were arranged in a single layer on perforated drying trays to ensure uniform air circulation. The sample load was fixed at 250 g for each drying run according to the dryer capacity and was kept constant throughout the experiments.

Based on preliminary trials, four drying temperatures (50°C, 60°C, 70°C, and 80°C) were selected to evaluate the effect of thermal treatment during convectional drying. Drying was initiated immediately after the trays were placed in the preheated dryer. Sample weight was monitored at regular intervals throughout the process, and the total drying time was recorded as the period required to reach the predefined drying endpoint. Drying was terminated when the samples reached a final moisture content of 10%, which was considered the drying endpoint. The water activity (aw) of the samples was subsequently measured using a water activity meter (LabSwift‐aw, Novasina, Switzerland) to verify that the samples had reached an aw value below 0.6.

Convectional drying (CD) experiments were conducted in two independent batches under identical operating conditions to account for batch‐to‐batch variability and to improve the reliability and reproducibility of the results (Karacabey et al. 2016).

After the drying process, the samples were stored in vacuum‐sealed polyethylene packaging at room temperature, and the packages were opened for analysis after all drying processes were completed.

### 2.3 Drying in the Microwave System (MD)

In this method, microwave (MJ32281BP; LG, Turkey) with different output power of 2450 MHz, 180–900 W was used. As a result of the preliminary experiments, four process parameters, 180, 360, 540, and 720 W, were determined for the microwave drying process.

According to the capacity of the microwave oven, 250 g of sample was placed in the microwave cavity. Cactus cladodes were sliced to a thickness of 5 mm and dried at microwave power levels of 180, 360, 540, and 720 W until a final moisture content of 10% was reached, which was considered the drying endpoint. The water activity (aw) of the dried samples was subsequently measured using a water activity meter (LabSwift‐aw, Novasina, Switzerland) to verify that the aw value was below 0.60. Microwave drying (MD) was performed in two independent drying runs (Karacabey et al. 2017). After the drying process, the samples were stored in vacuum‐sealed polyethylene packaging at room temperature, and the packages were opened for analysis after all drying processes were completed.

### 2.4 Ultrasound Pretreated Vacuum‐Assisted Convectional Drying (UVD)

Ultrasound pretreatment was performed using an ultrasonic processor (CY‐500; Optic Ivymen System, Spain) operating at a frequency of 20 kHz and equipped with a titanium alloy probe. The probe was immersed in 800 mL of distilled water contained in a 1000‐mL glass beaker. Cactus cladode slices (200 g) were used in all experiments, and the sample load was kept constant throughout the study.

Sonication was carried out at 100% amplitude for 30 min. The actual acoustic power delivered to the liquid medium was determined by the calorimetric method, as described by Raso et al. (1999), Kobus and Kusinska (2008), and Vartolomei et al. (2022). The measured acoustic power transferred into the medium was 8.60 W.

Based on the probe geometry (5.6 mm diameter), the acoustic intensity was calculated as 350 kW/m^2^, and the specific energy input (SEI) was 19.35 MJ/m^3^ (J/mL). The temperature of the medium was continuously monitored during sonication; the initial temperature ranged between 18°C and 19°C and did not exceed 23°C at the end of the treatment.

Following ultrasound pretreatment, vacuum drying was performed at 50°C, 60°C, 70°C, and 80°C in a vacuum oven until the samples reached a final moisture content of 10%, which was considered the drying endpoint. The water activity (aw) of the samples was subsequently measured using a water activity meter (LabSwift‐aw, Novasina, Switzerland) to verify that the aw value was below 0.60 (Tekin Cakmak et al. 2021).

After the drying process, the samples were stored in vacuum‐sealed polyethylene packaging at room temperature, and the packages were opened for analysis after all drying processes were completed.

### 2.5 Moisture Content Determination

Moisture content was determined using a halogen moisture analyzer (KERN DBS 60‐3, Germany) operating in rapid drying mode, based on a thermogravimetric method according to AOAC Official Method 925.10. Approximately 2–5 g of sample was evenly distributed on the sample pan and dried at 105°C until constant weight was reached. Moisture content was calculated as percentage weight loss and expressed on a dry matter basis (DM) (AOAC International 2005; Lee 1995). Data are expressed as the mean ± standard deviation (SD) based on two independent experimental batches with three analytical replicates per batch.

### 2.6 Color Measurement

Color measurements of fresh, dried samples were carried using High‐Quality Colorimeter (3nh, China). Color measurements are expressed with the CIE (L*, a*, b*) color system. In addition, total color difference (Δ*E*), Chroma (*C*), and Hue angle (*h*°) were calculated using Equations ((1), (2), (3)). L*, a*, and b* parameters were used as key indicators in evaluating color changes occurring during the drying process:

(1) $$∆E=\sqrt{{\left(L_{0}^{*}–L^{*}\right)}^{2}+{\left(a_{0}^{*}-a^{*}\right)}^{2}+{\left(b_{0}^{*}-b^{*}\right)}^{2}}$$

(2) $$\text{Chroma}=\sqrt{\left(a^{*2}+b^{*2}\right)}$$

(3) $$\mathrm{Hue}\;{\text{agle}}^{{}^{\circ}}=\tan^{-1}\left(b^{*}/a^{*}\right),$$

where subscript “0” refers to the color reading of fresh samples. Fresh samples were used as the reference and a larger Δ*E* denotes greater color changes (Dadali et al. 2007). Data are expressed as the mean ± standard deviation (SD) based on two independent experimental batches with three analytical replicates per batch.

### 2.7 Textural Properties

Texture properties of fresh and dried samples were evaluated using a texture analyzer (Stable Micro Systems, TA.XTplus, UK). A puncture (drilling) test was performed using a cylindrical stainless‐steel probe (P/2, 2 mm diameter, 26 mm usable length).

The probe was selected due to the heterogeneous structure of cactus cladode tissues to ensure localized penetration and to capture both compressive and shear resistance. Samples were cut into uniform slices. Fiber orientation was kept consistent by positioning samples so that the longitudinal fibers were perpendicular to the probe movement.

Test conditions were set as follows: test speed 1.0 × 10^−3^ m/s and strain 300%. For each drying condition, two independent drying runs were performed. From each independent drying run, three samples were evaluated for textural properties, and five instrumental measurements were performed for each sample. The five instrumental measurements were averaged to obtain a single value for each sample. The three sample values were then averaged to obtain the analytical mean for each independent drying run. The two independent drying runs were considered as the experimental units for statistical analysis (*n* = 2). Textural properties were expressed as skin strength (N) and elasticity (mm) according to Sirisomboon et al. (2012).

Both mean values and standard deviations were converted from gram‐force to Newton using a constant conversion factor (1 gf = 0.00981 N). This linear transformation does not affect statistical significance.

### 2.8 Extraction of Plant Material

The extraction process was performed according to the methodology defined by Uma et al. (2010). The fresh cactus cladode sample (FS) and dried samples were subjected to the extraction process.

Around 2 g of fresh or dried sample was ground and transferred to glass bottles and 20 mL of methanol was added. It was homogenized for 1 min using an ultraturrax device. The homogenized cladode sample was magnetically stirred on a magnetic stirrer for 120 min by wrapping aluminum foil at room temperature. The sample taken from the glass bottle was filtered through filter paper. Centrifugation of the filtrate was performed at 4000 rpm (≈419 rad/s) for 10 min under refrigerated conditions (4°C). The obtained extract was used for further analysis. Data are expressed as the mean ± standard deviation (SD) based on two independent experimental batches with three analytical replicates per batch.

### 2.9 Total Phenolic Content

The total phenolic content of all samples was quantified using the Folin–Ciocalteu assay in accordance with the procedure reported by Matthäus (2002). Spectrophotometric readings were obtained at a wavelength of 765 nm using a T70+ UV/Vis spectrophotometer. Quantification was carried out using a gallic acid calibration curve, and the results were expressed as milligrams of gallic acid equivalents per gram of dry matter (mg GAE/g DM), where GAE refers to gallic acid equivalents and DM refers to dry matter (Nikolaeva et al. 2022). Data are expressed as the mean ± standard deviation (SD) based on two independent experimental batches with three analytical replicates per batch.

### 2.10 Determination of Antioxidant Activities

Antioxidant potential of cladode samples was determined by two methods:

#### 2.10.1 2,2‐Diphenyl‐2‐picrylhydrazyl (DPPH˙) Assay

The total antioxidant capacity (TAC) of the samples was determined based on the method described by Dorman et al. (2003). Absorbance measurements were performed at a wavelength of 515 nm using a UV/VIS spectrophotometer. The inhibition percentages of the samples were calculated using Equation (4).

(4) $$\%\text{DPPH}˙=\left(A_{\text{control}}-A_{\text{sample}}\right)/A_{\text{control}}\times 100$$

where *A*_control_ is the absorbance of control (only DPPH˙ without extract) and *A*_sample_ is the absorbance of sample extract. Calibration was performed with Trolox solution and expressed as micromoles of Trolox equivalent per gram of dry matter (μmol TE/g DM). Data are expressed as the mean ± standard deviation (SD) based on two independent experimental batches with three analytical replicates per batch.

#### 2.10.2 2,2′‐Azino‐bis 3‐ethylbenzothiazoline‐6‐sulphonic Acid (ABTS^˙+^) Assay

The ABTS method developed by Re et al. (1999) was used to determine the antioxidant activities of fresh and dried cladodes. The ABTS˙^+^ radical solution was diluted with absolute ethanol to achieve an absorbance value of 0.70 ± 0.02 at a wavelength of 734 nm. The calibration curve was prepared using Trolox, a water‐soluble vitamin E derivative, and the results were expressed in micromole Trolox equivalent per dry matter (μmol TE/g DM). Data are expressed as the mean ± standard deviation (SD) based on two independent experimental batches with three analytical replicates per batch.

### 2.11 Total Flavonoid Content

The methanol content of the extracts was completely evaporated in a rotary evaporator and the extracts were completed with ethanol in accordance with this analysis. Total flavonoid levels in fresh and dried samples were determined using the method of Zaporozhets et al. (2004), employing rutin as the calibration standard, and spectrophotometric readings were taken at 510 nm. The calibration curve was created using standard rutin ethanolic solution. The flavonoid content was expressed as mg rutin equivalents per gram (mg RE/g DM). Data are expressed as the mean ± standard deviation (SD) based on two independent experimental batches with three analytical replicates per batch.

### 2.12 Total Flavonol Content

For total flavonol analysis, methanolic extracts were first obtained from the samples. The solvent was completely removed using a rotary evaporator (Heidolph Hei‐VAP, Heizbad, Germany), and the dried extracts were redissolved in ethanol prior to analysis. Total flavonol content of fresh, dried, and dried‐stored samples was determined according to the method described by Yermakov et al. (1987). Absorbance measurements were carried out at 440 nm using a UV/Vis spectrophotometer (T70+; PG Instruments, UK). Prior to spectrophotometric analysis, samples were filtered through filter paper to remove particulate matter.

A calibration curve was constructed using rutin as the standard in ethanolic solution, and total flavonol content was expressed as milligrams of rutin equivalents per 100 g of dry matter (mg RE/100 g DM). Data are expressed as the mean ± standard deviation (SD) based on two independent experimental batches with three analytical replicates per batch.

### 2.13 Phenolic Profile Analysis

Phenolic profile of fresh and dried samples chromatographically by HPLC (LC‐20ad pump, SPDM20A DAD detector, SIL‐20A HT autosampler, CTO‐10ASVP column oven, DGU‐20A5R degasser and CMB‐20A communication bus module) (HPLC‐DAD; Shimadzu Corp., Kyoto, Japan) system. Phenolic profile of fresh and dried samples Karadag et al. (2020) according to the method applied. One gram of the samples was weighed, divided into small pieces, transferred to glass bottles with 16 mL of methanol and extracted for 2 h in a magnetic stirrer by putting cactus cladode in it. The methanolic extracts were clarified using a 0.45 μm membrane filter, after which aliquots of 1 mL were collected in vials for system injection. Compounds were identified and quantified using retention time comparison and standard calibration curves. Details of the HPLC operating parameters and the gradient program are provided in Tables 1 and 2, respectively. Phenolic compounds were detected at multiple wavelengths, specifically 278, 320, and 360 nm, depending on the absorption characteristics of the target compounds. Quantitative data are reported as mg/g DM.

**TABLE 1:** HPLC operating conditions of phenolic profile analysis.

System	Working conditions
Device brand	Shimadzu
Device model	LC‐20AD
Column	Inertsil ODS C‐18 (250 × 4.6 mm, ID: 5 μm)
Detector	SPDM20A DAD
Mobile phase	0.1% Acetic acid (v/v), 0.1% Acetonitril (v/v)
Column temperature	40°C
Detection	278, 320 ve 360 nm
Flow rate	1 mL/min
Flow time	63 min

**TABLE 2:** HPLC gradient program.

Time (min)	Mobile phase‐acetonitril (%)
0	10
2	10
27	30
50	90
60	100
63	10

### 2.14 Ascorbic Acid Content

The ascorbic acid content of fresh, dried samples was determined chromatographically using HPLC. It was determined according to the method modified by Demiray et al. (2013). Three grams of sample was taken and diluted with 4.5% metaphosphoric acid at a ratio of 1:12.5 (g:mL) and homogenized. The sample was subsequently subjected to centrifugation at 4000 × *g* for 20 min at 4°C, after which the supernatant was clarified using a 0.45 μm Millipore filter. Prepared sample was automatically injected at a volume of 20 μL. All analyzes were performed in duplicate. HPLC operating conditions are shown in Table 3. Mobile phase was isocritically flowed. The amount of L‐ascorbic acid of the samples was prepared with standard ascorbic acid solution and determined with a standard curve. Results were expressed as milligrams of ascorbic acid per gram of dry matter (mg ascorbic acid/g DM), where DM (dry matter) represents the mass of the sample after complete removal of moisture.

**TABLE 3:** HPLC operating conditions.

System	Working conditions
Device brand	Agilent
Device model	1260 Infinity
Column	Agilent XBD C18 (250 × 4.6 mm, 5 μm)
Detector	DAD
Mobile phase	Ultrapure water
Column temperature	30°C
Detection	254 nm
Flow rate	0.8 mL/min
Flow time	35 min

### 2.15 Scanning Electron Microscopy (SEM) Analysis

All samples were lyophilized prior to scanning electron microscopy (SEM) analysis. To observe the structural changes as a result of the different drying methods and drying conditions, the samples were identically prepared (same size). Thin slices of about 1 mm thick were cut from the fresh and dried samples. Microstructural examination of cactus cladode slices, both fresh and dried, was performed by scanning electron microscopy using a Large Field Detector (LFD). As working conditions, low vacuum mode (70–71 Pa), mag 500× and 20 kV were used (Ikram et al. 2021).

### 2.16 Statistical Analysis

Statistical analyses were performed using SPSS statistical software (IBM, Armonk, NY, USA). The effects of drying treatments on the measured quality parameters were evaluated using one‐way analysis of variance (ANOVA), with drying treatment considered as a fixed factor. Each drying condition was performed in duplicate using two independent drying runs (*n* = 2). For each quality parameter, three analytical measurements were performed within each independent drying run and were considered technical replicates. The mean of the three technical replicates obtained from each independent drying run was calculated and used as the experimental value for statistical analysis. Thus, the independent drying run was considered the experimental unit, and the two independent run means (*n* = 2) were used for statistical analysis rather than treating the six analytical measurements as independent observations. The results were expressed as mean ± standard deviation (SD). When significant differences were detected by ANOVA (*p* < 0.05), Tukey's honestly significant difference (HSD) test was used for post hoc multiple comparisons among treatment means. Differences were considered statistically significant at *p* < 0.05.

## 3 Results and Discussion

According to the literature, interest in cactus cladodes as a component of the human diet has increased considerably in recent years and is expected to continue growing. Although several studies have reported on the drying of cactus cladodes, comprehensive information regarding the combined effects of different drying technologies and drying conditions on their quality characteristics remains limited. In particular, comparative data on the physicochemical properties, bioactive compound retention, antioxidant activity, and functional characteristics of dried cladodes are still scarce. Therefore, the present study aimed to evaluate the effects of different drying methods and drying conditions on cactus cladodes and to assess the resulting changes in their physicochemical properties, bioactive compounds, and functional attributes during storage.

### 3.1 Physicochemical Analysis

As can be seen in Table 4, the amount of dry matter of the studied sample was increased with the drying process (*p* < 0.05). In addition, drying time decreased with an increase in temperature in a convectional drying process (CD) or in an ultrasound‐pretreated vacuum‐assisted convectional drying technique (UVD), or with an increase in power level used in a microwave drying process (MD) (*p* < 0.05).

**TABLE 4:** Physicochemical properties of fresh and dried samples.

Samples	Dry matter (%)	Drying time (min)	Chromatic parameters						Textural properties	
			∆*E*	L*	a*	b*	Chroma	Hue°	Skin strength (N)	Elasticity (mm)
FS	7.75 ± 0.01 c	—	—	42.42 ± 10.16 ab	2.04 ± 3.61 c	23.44 ± 4.66 ab	28.17 ± 4.90 a	94.5 ± 9.3 a	1.836 ± 0.001 bcd	2.61 ± 0.01 bc
50°C CD	89.17 ± 0.01 ab	270 ± 1.41 d	14.82 ± 6.73 ab	36.31 ± 8.38 ab	0.01 ± 2.16 bc	15.77 ± 3.20 b	15.77 ± 10.35 a	89.9 ± 7.8 ab	4.346 ± 0.437 bcd	2.70 ± 0.15 bc
60°C CD	89.12 ± 0.01 ab	210 ± 1.41 f	14.82 ± 4.03 ab	36.76 ± 7.03 ab	0.20 ± 1.53 bc	15.55 ± 1.00 b	15.55 ± 1.30 a	89.2 ± 1.20 ab	3.896 ± 0.800 bcd	1.81 ± 0.12 c
70°C CD	91.03 ± 0.01 a	180 ± 1.41 g	11.19 ± 0.67 ab	39.33 ± 0.77 ab	0.82 ± 0.43 bc	17.62 ± 1.00 ab	17.64 ± 2.40 a	92.6 ± 4.65 ab	4.649 ± 0.465 bcd	2.27 ± 0.91 bc
80°C CD	94.59 ± 0.01 a	165 ± 2.12 h	20.45 ± 4.60 ab	29.76 ± 2.70 ab	1.64 ± 0.64 bc	12.90 ± 3.70 b	13.00 ± 0.90 a	82.7 ± 5.79 ab	3.563 ± 0.370 bcd	2.98 ± 0.33 bc
180 W MD	87.06 ± 0.01 ab	90 ± 1.41 i	17.54 ± 5.48 ab	31.20 ± 7.22 ab	5.58 ± 0.41 abc	17.33 ± 4.80 ab	18.21 ± 7.70 a	72.1 ± 1.96 abc	4.001 ± 0.200 bcd	3.15 ± 0.17 bc
360 W MD	89.12 ± 0.01 ab	60 ± 1.41 j	18.43 ± 3.51 ab	31.20 ± 3.24 ab	7.02 ± 0.83 abc	17.48 ± 1.80 ab	18.83 ± 1.99 a	68.1 ± 1.58 bc	2.855 ± 0.404 bcd	2.48 ± 1.15 bc
540 W MD	90.21 ± 0.01 ab	30 ± 2.12 k	19.88 ± 0.04 ab	30.13 ± 0.85 ab	5.64 ± 0.82 abc	15.20 ± 1.35 ab	16.21 ± 1.77 a	69.6 ± 1.49 bc	4.125 ± 0.018 bcd	2.89 ± 0.90 abc
720 W MD	90.10 ± 0.01 ab	20 ± 2.12 L	7.94 ± 0.23 b	43.07 ± 0.55 ab	5.06 ± 0.24 abc	25.79 ± 0.08 ab	26.29 ± 7.88 a	78.9 ± 5.69 abc	4.161 ± 0.266 bcd	2.46 ± 0.16 bc
50°C UVD	81.29 ± 0.01 b	1080 ± 3.54 a	11.72 ± 4.46 b	43.67 ± 4.56 ab	7.60 ± 3.30 abc	28.92 ± 6.87 a	29.90 ± 2.43 a	75.2 ± 2.14 abc	7.586 ± 1.761 abcd	6.14 ± 2.76 abc
60°C UVD	81.63 ± 0.01 b	780 ± 3.54 b	11.19 ± 1.65 b	46.53 ± 5.73 a	3.90 ± 0.78 bc	21.93 ± 2.61 ab	22.27 ± 10.08 a	79.9 ± 4.47 abc	14.076 ± 3.972 a	10.54 ± 2.00 a
70°C UVD	84.21 ± 0.01 ab	420 ± 3.54 c	19.18 ± 6.47 ab	39.85 ± 14.47 ab	8.89 ± 6.15 ab	22.59 ± 5.75 ab	24.28 ± 6.41 a	68.5 ± 10.33 bc	11.676 ± 7.904 ab	8.57 ± 3.97 a
80°C UVD	92.62 ± 0.01 ab	240 ± 3.54 e	23.62 ± 6.71 a	28.75 ± 5.94 b	14.70 ± 0.64 a	25.55 ± 5.30 ab	29.48 ± 7.29 a	60.0 ± 13.0 c	11.445 ± 3.776 abc	7.61 ± 2.79 ab

*Note:* The results were expressed as means ± SD. Means sharing different lowercase letters within the same column indicate statistically significant differences among treatments (*p* < 0.05).

Abbreviations: CD, convectional drying; DM, dry matter; FS, fresh sample; MD, microwave drying; UVD, ultrasound‐pretreated vacuum‐assisted convectional drying.

Color, which is considered as a physicochemical property of the product, is one of the most important quality parameters of dried products (Ozkan et al. 2003). It is also a critical factor affecting the acceptance of the product by the consumer. The color values of the samples are shown in Table 4. L* illuminance (brightness) values of no statistically significant differences were found between the samples, a general trend observed in fresh cladodes (*p* > 0.05). However, L* values of samples dried at 60°C by UVD were higher than L* values of samples processed by the same technique, but at a temperature level of 80°C (*p* < 0.05). The increase in L* values may be attributed to ultrasound‐induced cell rupture and the formation of a more porous microstructure, which enhances light scattering and results in a brighter appearance. This structural effect was more pronounced at 60°C, where thermal degradation reactions were relatively limited, allowing the light‐scattering effect to dominate.

At higher temperatures (70°C–80°C), however, thermal processes such as chlorophyll degradation, nonenzymatic browning reactions, and pigment oxidation likely became more dominant, masking the structural effect of ultrasound and resulting in lower L* values (Cao, Islam, et al. 2020; Wang et al. 2026).

As a general trend, drying techniques did not cause any statistically significant change on a* values compared to each other (*p* > 0.05).

The highest a* values belong to the samples processed by UVD. Among these samples, the a* value of the dried sample at 80°C by UVD is significantly higher than the samples obtained by CD and the control sample (*p* < 0.05). The higher a* values observed in UVD samples may be attributed to the interaction between ultrasound treatment and drying temperature. This change in a* values may be attributed to thermal reactions in chemical color pigments. Chlorophyll components in the fresh sample are damaged by drying processes. Similarly, López‐Cervantes et al. (2011) dried cactus cladodes at 60°C, 70°C, and 80°C. It was stated that the a* value increased and the greenness in the product color decreased with the increase in drying temperature. This change was attributed to the thermal conversion of chlorophyll into pheophytin, which is associated with the displacement of magnesium from the chlorophyll molecule during drying (López‐Cervantes et al. 2011). Addition, Nguyen et al. (2023) stated that increasing drying temperature and time can cause enzymatic and nonenzymatic reactions in the product, leading to chlorophyll degradation and changes in color values.

As seen in Table 4, there was no significant change in the b* values of the dried samples compared to the fresh samples (*p* > 0.05).

The total color change value (∆E) was also calculated compared to fresh samples. The results showed that there were significant changes in ∆*E* values with drying processes (*p* < 0.05).

Considering the ∆*E* values, the highest difference with the fresh sample was observed in the UVD at 80°C (23.62). Again, it was determined that the ∆*E* value of this application was higher than dried samples with 50°C and 60°C by UVD and the samples at 720 W power MD (*p* < 0.05). There was no difference between the other drying samples (*p* > 0.05).

In addition, relatively low Δ*E* values were observed in the 720 W MD and 70°C CD treatments, indicating a lower overall color change and better color retention in these samples compared to the other drying conditions.

Similar results have been obtained by different researchers. Guiné and Barroca (2012) stated that the ∆*E* values of zucchini and green peppers dried with convection drying technique at 30°C and 70°C increased with the increase in drying temperature. Aamir and Boonsupthip (2017) reported an increase in ∆*E* values as microwave power increased. Tekin and Baslar (2018) applied different drying techniques (45°C, 55°C, 65°C, 75°C ultrasound‐pretreated vacuum‐assisted convectional drying and convectional drying) to red peppers. The lowest ∆*E* values were obtained in ultrasound‐pretreated vacuum‐assisted convectional drying technique and at low temperatures. In that study, total color changes were associated with carbohydrates and proteins which were affected by reactions as a result of high process temperatures and the effect of oxygen presence.

Chroma value, another color parameter, was also examined. However, the drying process did not create any statistically significant differences in Chroma values of samples depending on the methods applied or the process conditions used in those methods (*p* > 0.05) (Table 4).

The Hue value, that is, the angle of the color, changes according to the point where the color is red, green and blue. Hue values range from 0°, which is red, to 90°, which is yellow; 180°, which is green; and 270°, which is blue. The fresh sample's interior structure's hue value was determined to be 94.59°. Except for a few cases, the drying procedure had no effect on hue value (*p* > 0.05). Only samples dried by UVD at 70°C and 80°C and by MD at 360 and 540 W significantly differed from fresh samples in terms of the reduction in hue value (*p* < 0.05). With these applications, it was noticed that the fresh product's original green color eventually turned yellow. The sample dried by UVD at 80°C (60.09°) has the lowest Hue value. This application showed the most noticeable color change from green to yellow (*p* < 0.05) (Table 4).

Maskan (2001) subjected the kiwi fruit to hot air drying and microwave drying processes. Similar to our results, both drying techniques showed decreases in Chroma and Hue values compared to the fresh sample. With the increase in drying time, the product's color changed to green, yellow, and red.

Texture is another quality parameter that determines the acceptability of dried food products by consumers (Perera 2005). Two important textural parameters of the samples, skin strength and elasticity, are tabulated in Table 4. As seen in Table 4, the change in the skin strength value of the product with drying processes was statistically insignificant in CD and MD applications (*p* > 0.05). Xie et al. (2023) stated that there was no difference in hardness values between peanuts dried using hot air drying and those dried using vacuum drying. Addition, Jafari et al. (2016) applied convectional drying of kiwi and reported similar findings for product firmness.

The change in the skin strength value of the product was found to be significant in drying performed at 60°C with UVD method (*p* < 0.05) (Table 4). An increase in skin strength can be associated with the removal of moisture (Kotwaliwale et al. 2007). Similar results to our study were also reported by different authors. Deng and Zhao (2008) showed that ultrasound pretreatment increased firmness in air‐dried and freeze‐dried apples. With the effect of ultrasound, the cell wall was damaged, its contents were released to the medium; thus, the water transfer increased.

Compared to the fresh sample, the highest elasticity values were observed in the samples dried at 60°C and 70°C with UVD method (*p* < 0.05) (Table 4). Cell walls were damaged by the ultrasound effect, cell contents were transferred to the solvent in the medium, and cell water transfer increased.

### 3.2 Total Phenolic, Flavonoid, Flavonol, and Ascorbic Acid (Vitamin C)

Ascorbic acid and phenolic compounds are among the most important natural antioxidants found in plant and animal sources. Polyphenolic compounds are classified into various categories based on the number of phenolic rings in their molecular structure and the type of bonding between these rings. These compounds are primarily grouped as phenolic acids, flavonoids, stilbenes, and lignans. The flavonoid group, in turn, consists of six main subclasses: flavonols, flavones, isoflavones, flavanones, anthocyanidins, and flavanols (catechins and proanthocyanidins). These compounds act as antioxidants, counteract oxidative stress, and modulate the activity of a wide variety of enzymes and receptor cells (Manach et al. 2004).

As seen in Table 5, the drying process led to a significant reduction in phenolic compounds (*p* < 0.05). During the CD process, a decrease in phenolic content was seen (*p* < 0.05) by the temperature change from 70°C to 80°C. Similarly, Chan et al. (2009) showed that drying treatments using microwave, convectional oven, and solar energy resulted in significant reductions in total polyphenol levels in the leaves of *Alpinia zerumbet* , *Etlingera elatior* , *Curcuma longa* , and *Kaempferia galanga* . Also, López et al. (2010) reported that a significant amount of total polyphenol substance loss occurred in blueberries as the convectional drying temperature increased. Similarly, de Figueiredo et al. (2024) dried broccoli leaves at 50°C, 60°C, and 70°C. Increasing the drying temperature resulted in a decrease in phenolic compound values.

**TABLE 5:** Total phenolic, flavonoid, flavanol, and ascorbic acid (vitamin C) results of fresh and dried samples.

Samples	Spectrophotometric assays			Chromatographic assay
	Total phenolic (mg GAE/g DM)	Flavonoid (mg rutin/g DM)	Flavonol (mg/100 g DM)	Ascorbic acid (mg/g DM)
FS	8.85 ± 1.12 a	1.97 ± 0.01 abc	2.43 ± 9.82 a	0.468 ± 0.006 a
50°C CD	3.18 ± 0.05 def	2.86 ± 0.06 a	0.49 ± 0.11 b	0.465 ± 0.004 a
60°C CD	2.75 ± 0.07 ef	2.62 ± 0.18 ab	0.39 ± 0.07 b	0.216 ± 0.011 b
70°C CD	3.11 ± 0.38 ef	1.84 ± 0.26 abcd	0.31 ± 0.06 b	0.182 ± 0.002 d
80°C CD	2.16 ± 0.25 f	2.11 ± 0.56 abc	0.28 ± 0.03 b	0.094 ± 0.003 e
180 W MD	3.88 ± 0.35 cde	2.94 ± 0.41 a	0.42 ± 0.03 b	0.176 ± 0.003 d
360 W MD	3.41 ± 0.23 def	2.31 ± 1.85 abc	0.34 ± 0.08 b	0.087 ± 0.002 e
540 W MD	3.22 ± 0.32 def	2.18 ± 0.69 abc	0.38 ± 0.21 b	0.012 ± 0.001 h
720 W MD	4.41 ± 0.37 bcd	2.84 ± 0.12 a	0.36 ± 0.07 b	0.195 ± 0.003 bc
50°C UVD	2.26 ± 0.26 f	1.06 ± 0.08 cde	0.46 ± 0.21 b	0.190 ± 0.001 cd
60°C UVD	4.97 ± 0.44 bc	1.28 ± 0.01 bcde	0.64 ± 0.03 b	0.008 ± 0.000 h
70°C UVD	5.30 ± 0.01 b	0.50 ± 0.07 e	0.75 ± 0.04 b	0.030 ± 0.000 g
80°C UVD	2.63 ± 0.27 f	0.53 ± 0.53 de	0.68 ± 0.01 b	0.047 ± 0.002 f

*Note:* The results were expressed as means ± SD. Means sharing different lowercase letters within the same column indicate statistically significant differences among treatments (*p* < 0.05).

Abbreviations: CD, convectional drying; DM, dry matter; FS, fresh sample; MD, microwave drying; UVD, ultrasound‐pretreated vacuum‐assisted convectional drying.

During the microwave drying process, as the microwave power increased from 180 to 540 W, the total phenolic component content decreased (*p* < 0.05). However, this trend was reversed by the power shift from 540 W (3.22 mg GAE/g DM) to 720 W (4.41 mg GAE/g DM) and the total phenolic content increased in these upper power levels (Table 5) (*p* < 0.05). As the power used in microwave drying increased, the drying time was shortened. This increase is suggested to be related to shorter drying times, more efficient release of phenolic compounds from the cellular matrix, and the formation of new compounds as a result of Maillard reactions (Inchuen et al. 2010). Indeed, the same researchers reported that increasing the microwave application power increased the total polyphenol content of red curry powder. Additionally, Salehi et al. (2023) stated that sweet cherry microwave treatment increased the phenolic compound content.

The phenolic contents of the microwave‐dried samples were higher than the convectional dried samples (*p* < 0.05) (Table 5). Similar results to the current ones have been reported in the literature. Hamrouni‐Sellami et al. (2013) determined that the phenolic content for sage sample dried with different drying techniques (conventional hot air and microwave drying), and the highest amount was found in sage plants dried by microwave. That favorable effect was associated with the heating mechanism of the microwave system. The intense heat generated by microwaves creates a high vapor pressure, and the temperature inside the plant tissue causes the plant cell wall polymers to break down, making more phenolic extractable (Hamrouni‐Sellami et al. 2013).

Gouws et al. (2019) investigated the effect of different drying techniques on the physicochemical and antioxidant properties of cactus fruit and stated that the phenolic compound content of the samples dried with the microwave drying technique was higher than that of the samples dried in the convectional oven.

The UVD samples, especially those dried at 60°C and 70°C (4.97, 5.30 mg GAE/g DM), showed higher total phenolic content compared to the CD (2.75, 3.11 mg GAE/g DM) samples (*p* < 0.05) (Table 5). Similar results have been reported by Yue et al. (2023) and Cao, Islam, et al. (2020). The reason for this increase may be related to the production of hydroxyl radicals, which could lead to hydroxylation of the aromatic ring of phenolic compounds at ortho‐, meta‐, and para‐positions (Ordóñez‐Santos et al. 2017). Another reason for the increase in phenolic component content with ultrasound pretreatment was determined by Yilmaz et al. (2021) and Higuera‐Orbe et al. (2021). The sound waves sent to the sample through pure water resulted in the vibration of the molecules and caused consecutive compressions and expansions among the molecules in the process environment. As a result of compression and expansion effects, gas bubbles were formed. These gas bubbles burst, creating cavitations. Thus, deterioration in the structure of the cell wall occurred and the substances in the cell were made free and it became easier to extract them. Similar results were reported by Turan et al. (2023).

In our study, it was determined that the total flavonoid content of the samples obtained at 70°C and 80°C in UVD process was lower than fresh and all other drying methods (*p* < 0.05) (Table 5). The reason for the loss observed here is thought to be the increase in temperature and drying time. A similar effect was found in de Albuquerque et al. (2021).

When comparing convection drying and vacuum drying performed at the same temperature, flavonoid contents are lower in the vacuum drying process. Similar results were obtained by Nguyen et al. (2023). It was observed that the flavonoid content slightly increased when the temperature increased from 70°C to 80°C in the CD process. This may be because the hot air used in the CD method promotes surface hardening of the sample, thus preventing excessive degradation of the flavonoids during this process (Vega‐Galvez et al. 2023). The same trend was also valid when the power level of the microwave oven was increased from 540 to 720 W (*p* < 0.05) (Table 5). Similar results were reported for total phenolic content of orange peels and leaves by Kammoun Bejar et al. (2011). These researchers subjected peels and leaves to microwave drying at different power levels, and when it was gradually shifted from 180 to 450 W, total phenolic content of dried materials was reached to its highest level in the 450 W application. This phenomenon was explained by the reduced application time and the structure of the fiber matrix becoming larger and looser, facilitating subsequent solvent extraction.

It was determined that the amount of flavonoids decreased in UVD at 70°C and 80°C (*p* < 0.05) (Table 5). It is thought that this reduction may be possible due to the transition of free flavonoid compounds to water medium during ultrasound pretreatment. There are also studies in the literature supporting our results. In a study examining carrots, spinach, potatoes, and various greens, it was stated that boiling and long‐term freezing storage reduced their total phenolic content by 20%–30%. In another study, it was stated that after spinach was boiled in water, half of the flavonoids were found in water and the other half in boiled spinach. In another study, the effects of different treatments applied to onions and tomatoes on the amount of quercetin flavonoid were examined, and it was stated that boiling resulted in 80% loss and microwave application resulted in 65% loss (Çapanoğlu and Boyacıoğlu 2009).

Changes in flavonoid content can also be explained by oxidation, polymerization, and decomposition reactions (Qiao et al. 2014). The number of bubbles formed during cavitation is temperature dependent, and temperature increases cell disruption. Therefore, the flavonoid content may increase. However, depending on the power of ultrasound, oxidation of phenolic substances occurs, and accordingly, their contribution to the antioxidant capacity may decrease considerably (Teh and Birch 2014; Bursać Kovačević et al. 2019). Solvent type and temperature play a major role in the flavonoid content, and their decomposition rate also depends on the ultrasound intensity. Li et al. (2019) *Psidium guajava* leaves were subjected to ultrasonic extraction using distilled water using different parameters. These researchers reported that the diffusion and solubility of flavonoids in water increase with the extraction temperature, but much higher temperatures may degrade some heat‐sensitive compounds, and similarly, when excessively high ultrasonic power is used, the cavitation bubble caused by ultrasound may prevent the transmission of ultrasonic waves and deform the structure of flavonoids.

As seen in Table 5, the amount of ascorbic acid (vitamin C) decreased as the CD temperature increased. This change was statistically significant (*p* < 0.05). Different researchers in the literature have also observed the same trend. Kaya et al. (2010) stated that increasing relative humidity and product temperature in the CD process increased the degradation of vitamin C in the product. Similarly, Khraisheh et al. (2004) applied CD and MD to dry potato samples. The effects of different CD temperatures (30°C, 40°C, and 60°C) and different MD power (10.5, 15, and 38 W) on vitamin C were investigated. It was stated that the degradation of vitamin C increased with the temperature or microwave power elevation.

Again, as seen in Table 5, the vitamin C amounts of the dried samples by UVD are quite low compared to the convectional dried samples (*p* < 0.05). It was thought that one of the reasons for this situation might be due to the transition of ascorbic acid content into water during the ultrasound pretreatment as a result of its high solubility, as the sample was kept in distilled water for 30 min. Another mechanism causing loss in ascorbic acid was related to the elevated temperature level of the drying process.

de Albuquerque et al. (2021) applied ultrasound to cactus water and investigated its effect on bioactive compounds (C0; control, HP10; 10 min heat application; US10; 10 min ultrasound application; US20; 20 min ultrasound application, US40; 40 min ultrasound application). US40 beverage obtained 22.67% less ascorbic acid than C0 (*p* < 0.05). This event showed that ultrasound and exposure time to ultrasonic waves adversely affected the vitamin C compound concentration in the beverage. The degradation of ascorbic acid was attributed to the oxidation mechanisms produced by the acoustic cavitation phenomenon due to the high shear stress in the liquid medium. The formation and subsequent collapse of microbubbles promoted extreme levels of highly localized turbulence. At the same time, an increase in system temperature in the presence of oxygen and the formation of free radicals (H^+^ and OH) that support aerobic oxidation also caused vitamin C loss.

### 3.3 Phenolic Profile

Phenolic compounds are important secondary metabolites involved in defense and adaptation processes in plants. The biosynthesis of these compounds is dynamically controlled by environmental conditions and various stress stimuli. Therefore, various treatments applied before and after harvest can lead to an increase or decrease in the amount of phenolic compounds in fruits and vegetables. Preharvest factors include temperature, soil structure, light intensity, irrigation regime, and fertilization practices; while in the postharvest period, mechanical damage, storage temperature, atmospheric conditions, and light exposure play a decisive role. Phenolic compounds contribute to the maintenance of structural integrity in plants and undertake protective functions against ultraviolet (UV) radiation, biotic and abiotic stresses, pathogens, and herbivores (Laura et al. 2019). However, drying, one of the most widely used postharvest preservation methods, may adversely affect the quality of medicinal plants by causing changes in color and aroma. These changes are mainly attributed to enzymatic and nonenzymatic reactions occurring during the drying process, which can alter the composition and stability of bioactive compounds through phenolic degradation and oxidative reactions. As a result, the content of antioxidant bioactive constituents and the overall antioxidant capacity of the dried material may be reduced, thereby affecting its nutritional, functional, and medicinal value (Babaei Rad et al. 2025).

The phenolic component contents of fresh and dried samples are given in Table 6. The total phenolic component content was affected by the drying processes (*p* < 0.05). In the samples, the major component was ellagic acid. As a result of the analysis, nine phenolic components were determined; eight of them were phenolic acids (gallic acid, protocatechuic acid, syringic acid, ellagic acid, m‐coumaric acid, caffeic acid, p‐coumaric acid, ferulic acid) and one was flavonoid (chrysin).

**TABLE 6:** Phenolic profile results of fresh and dried samples.

Component (mg/g DM)	Fresh	Convectional drying				Microwave drying				Ultrasound‐pretreated vacuum‐assisted convectional drying			
		50°C	60°C	70°C	80°C	180 W	360 W	540 W	720 W	50°C	60°C	70°C	80°C
Gallic acid	n.d	n.d	n.d	n.d	n.d	n.d	0.400 ± 0.0002 a	n.d	0.389 ± 0.0007 b	n.d	n.d	n.d	n.d
Protocarechic acid	n.d	n.d	0.330 ± 0.006 a	n.d	n.d	n.d	n.d	n.d	n.d	n.d	n.d	n.d	n.d
Syringic acid	n.d	n.d	n.d	n.d	n.d	n.d	n.d	n.d	n.d	n.d	0.020 ± 0.000 a	n.d	n.d
Ellagic acid	3.988 ± 0.0034 a	0.419 ± 0.001 de	0.497 ± 0.0047 cd	0.595 ± 0.0069 b	0.510 ± 0.0006 c	0.48 ± 0.0013 cd	0.428 ± 0.002 cde	0.426 ± 0.0055 de	0.358 ± 0.003 e	0.150 ± 0.071 f	0.488 ± 0.017 cd	0.457 ± 0.004 cd	0.419 ± 0.002 de
m‐Coumaric	n.d	0.090 ± 0.001 a	0.057 ± 0.001 d	0.038 ± 0.000 e	0.087 ± 0.001 a	0.069 ± 0.001 c	0.077 ± 0.001 b	0.07 ± 0.001 b	0.090 ± 0.0003 a	n.d	0.066 ± 0.000 c	0.068 ± 0.001 c	0.062 ± 0.000 cd
Chrysin	n.d	0.007 ± 0.000 h	0.053 ± 0.000 g	0.057 ± 0.000 f	0.077 ± 0.000 d	0.078 ± 0.000 cd	0.079 ± 0.001 bc	0.075 ± 0.001 e	0.080 ± 0.00 b	0.084 ± 0.000 a	0.085 ± 0.000 a	0.084 ± 0.000 a	0.078 ± 0.001 cd
Caffeic Acid	2.196 ± 0.006 a	0.209 ± 0.002 cdef	0.177 ± 0.007 g	0.179 ± 0.005 g	0.228 ± 0.003 b	0.199 ± 0.002 ef	0.207 ± 0.005 def	0.206 ± 0.005 def	0.205 ± 0.007 def	0.217 ± 0.003 bcde	0.226 ± 0.005 bc	0.220 ± 0.005 bcd	0.198 ± 0.000 f
p‐Coumaric	0.265 ± 0.008 a	0.023 ± 0.000 b	0.016 ± 0.001 b	0.016 ± 0.000 b	0.018 ± 0.000 b	0.018 ± 0.000 b	0.015 ± 0.000 b	0.015 ± 0.000 b	0.015 ± 0.000 b	0.015 ± 0.000 b	0.017 ± 0.000 b	0.018 ± 0.000 b	0.018 ± 0.001 b
Ferulic acid	1.050 ± 0.070 a	0.095 ± 0.007 b	0.076 ± 0.005 b	0.068 ± 0.003 b	0.085 ± 0.004 b	0.085 ± 0.003 b	0.078 ± 0.003 b	0.074 ± 0.003 b	0.078 ± 0.004 b	0.078 ± 0.003 b	0.087 ± 0.004 b	0.084 ± 0.003 b	0.072 ± 0.002 b
Total	7.50 ± 0.001 a	0.836 ± 0.003 f	1.203 ± 0.008 c	0.948 ± 0.001 de	1.000 ± 0.003 d	0.938 ± 0.001 de	1.274 ± 0.031 b	0.863 ± 0.005 f	1.128 ± 0.016 c	0.488 ± 0.002 g	0.972 ± 0.006 d	0.925 ± 0.004 e	0.839 ± 0.001 f

*Note:* The results were expressed as means ± SD. Means sharing different lowercase letters within the same column indicate statistically significant differences among treatments (*p* < 0.05).

Abbreviations: CD, convectional drying; DM, dry matter; FS, fresh sample; MD, microwave drying; n.d, not detected; UVD, ultrasound‐pretreated vacuum‐assisted convectional drying.

The changes observed in the phenolic profile of fresh and dried samples can be attributed to the combined effects of thermal degradation, structural disruption of plant tissues, and changes in extractability induced by different drying processes. The significantly lower total phenolic content in dried samples compared to fresh material suggests that phenolic compounds are sensitive to processing conditions, particularly temperature and oxygen exposure.

In addition to overall losses, variations in individual phenolic compounds were observed among drying treatments. The fact that some phenolic compounds are detected only in dried samples can be explained by the release of bound phenolic compounds from the plant matrix due to cell wall disruption and structural degradation during processing. Moreover, moisture loss during drying may have led to concentration effects, improving the detectability of compounds present at low levels in fresh samples. The possibility that these compounds were present in fresh samples below the detection limit of the analytical method cannot be excluded.

Among the identified compounds, ellagic acid was the predominant phenolic in fresh samples; however, its content decreased significantly after drying, indicating its susceptibility to thermal and oxidative degradation (Garcìa et al. 2021).

It is thought that the variation of the presence of the components in the samples may be different due to reasons such as the exposure of the cladodes to sunlight and the difference in the degree of maturity of the cladodes (Table 6). Di Bella et al. (2022) detected a total of 17 phenolic compounds, including 10 phenolic acids and 7 flavonoids, in dried Sicilian cladodes. In addition, they stated that the content of phenolic compounds was affected by both the variety and the pruning season. In a different study, a total of 6 compounds were identified (El‐Mostafa et al. 2014). Figueroa‐Pérez et al. (2018) detected 15 phenolic acids and 13 flavonoids in convectional dried cactus cladodes (*O. ficus indica*, *Milpa Alta cv*.). They also stated that the phenolic profile was affected by the maturity stage of the cladodes. In our study, unlike the results of these studies, the chrysin component was determined (Table 6).

### 3.4 Antioxidant Capacity

In Figures 1 and 2, antioxidant capacity values of fresh and dried samples determined by DPPH and TEAC method are expressed graphically.

**FIGURE 1:**
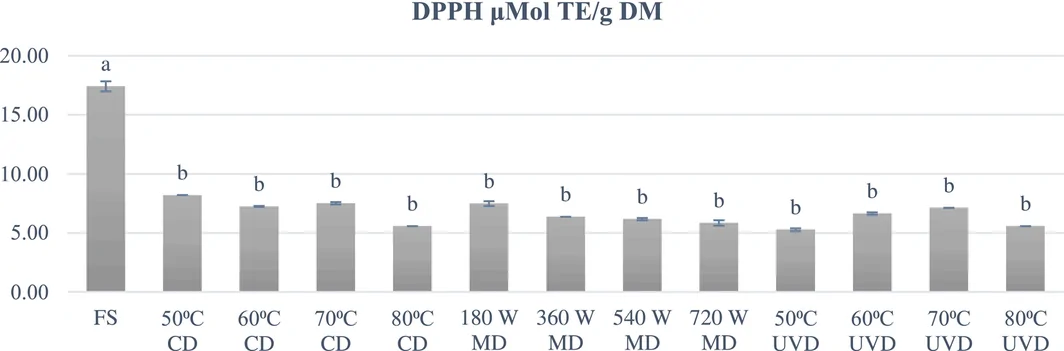
Antioxidant capacity of fresh and dried samples. The results were expressed as means ± SD. Means sharing different lowercase letters within the same column indicate statistically significant differences among treatments (*p* < 0.05). CD, convectional drying; DM, dry matter; FS, fresh sample; MD, microwave drying; UVD, ultrasound‐pretreated vacuum‐assisted convectional drying.

**FIGURE 2:**
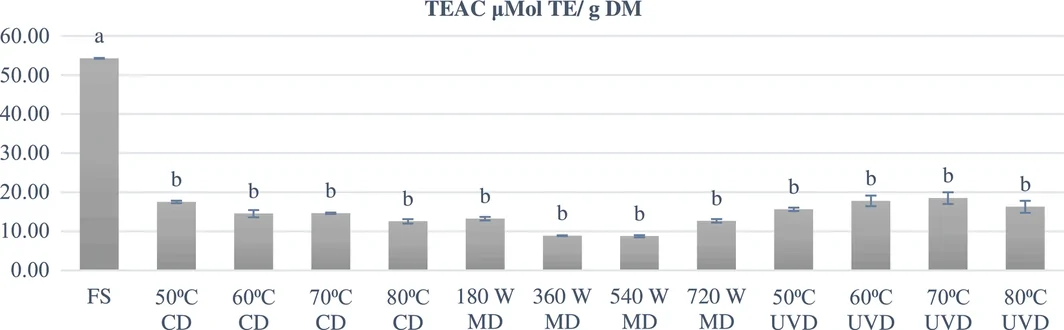
Antioxidant capacity of fresh and dried samples. The results were expressed as means ± SD. Means sharing different lowercase letters within the same column indicate statistically significant differences among treatments (*p* < 0.05). CD, convectional drying; DM, dry matter; FS, fresh sample; MD, microwave drying; UVD, ultrasound‐pretreated vacuum‐assisted convectional drying.

Antioxidant capacity results are 17.42, 8.20, 7.24, 7.51, 5.57, 7.47, 6.37, 6.16, 5.84, 5.27, 6.64, 7.11, and 5.57 μmol TE g^−1^ (FS, 50°C CD, 60°C CD, 70°C CD, 80°C CD, 180 W MD, 360 W MD, 540 W MD, 720 W MD, 50°C UVD, 60°C UVD, 70°C UVD, 80°C UVD) respectively, according to the DPPH method.

Antioxidant capacity results are 54.26, 17.52, 14.52, 14.62, 12.55, 13.24, 8.87, 8.74, 12.67, 15.62, 17.78, 18.51, and 16.27 μmol TE g^−1^ (FS, 50°C CD, 60°C CD, 70°C CD, 80°C CD, 180 W MD, 360 W MD, 540 W MD, 720 W MD, 50°C UVD, 60°C UVD, 70°C UVD, 80°C UVD) respectively, according to the TEAC method.

Considering the antioxidant capacity values, for both analysis methods, antioxidant capacity was lowered with the drying process irrespective of the technique used (*p* < 0.05), but there was no significant difference observed between drying techniques and the process conditions (*p* > 0.05). Similar results have been reported by Yuan et al. (2022) and Cao, Tao, et al. (2020). Although there were significant differences in total phenol content, no significant difference was observed in antioxidant capacity. Gallegos‐Infante et al. (2009) and explained that cactus cladodes are rich in monohydroxylated phenolic compounds and that such phenols provide lower antioxidant activity.

### 3.5 SEM (Scanning Electron Microscopy) Analysis

Effects of different drying methods and conditions on the microstructure of dried and fresh cactus cladode samples were observed by SEM method. As it can be seen in Figure 3, for the fresh sample, cactus cladode tissue is composed of many well‐arranged pores. When dried samples were examined, it was seen that after CD, cell volume was reduced and tissue was collapsed. And these cactus cladode samples had low porosity. Similar results were obtained by Celik and Parlak (2022) and Seremet et al. (2016). In the samples dried at 70°C, some serious tissue contractions were observed, which was quite evident, which no longer showed the vacuoles in the fresh sample inside the cell, and even brook the cell walls, due to the evaporation of water and uniting the cell walls. The collapse was thought to occur as a result of increased tissue dehydration with increased deformation of cell tissue and loss of cell turgor. Less microstructural changes were seen in microwave‐dried samples. The internal vapor pressure of water in these samples may have caused tissue expansion. This may be due to the lower shrinkage of microwave‐dried samples, shorter drying time due to the internal vapor pressure of the water, and tissue expansion. Also, increasing the microwave power level in microwave‐dried samples led to larger and wider gaps and deeper grooves in the mesocarp. The increased microwave power level had a more destructive effect on the cell walls than the drying time, because the microwave power level caused more heat generation inside the product. In addition, increasing the power level in the microwave drying process created higher pressure by providing faster evaporation within the product, thus opening larger pores and channels (Askari et al. 2004; Miraei Ashtiani et al. 2018; Cruz‐de la Cruz et al. 2020). Therdthai and Zhou (2009)'s results support our study. The change in the texture of the dried products UVD is shown in the Figure 3. In these examples, cell structures and interspaces are more prominent, especially at low temperatures. This situation is thought to be related to the sound waves in the ultrasound application.

**FIGURE 3:**
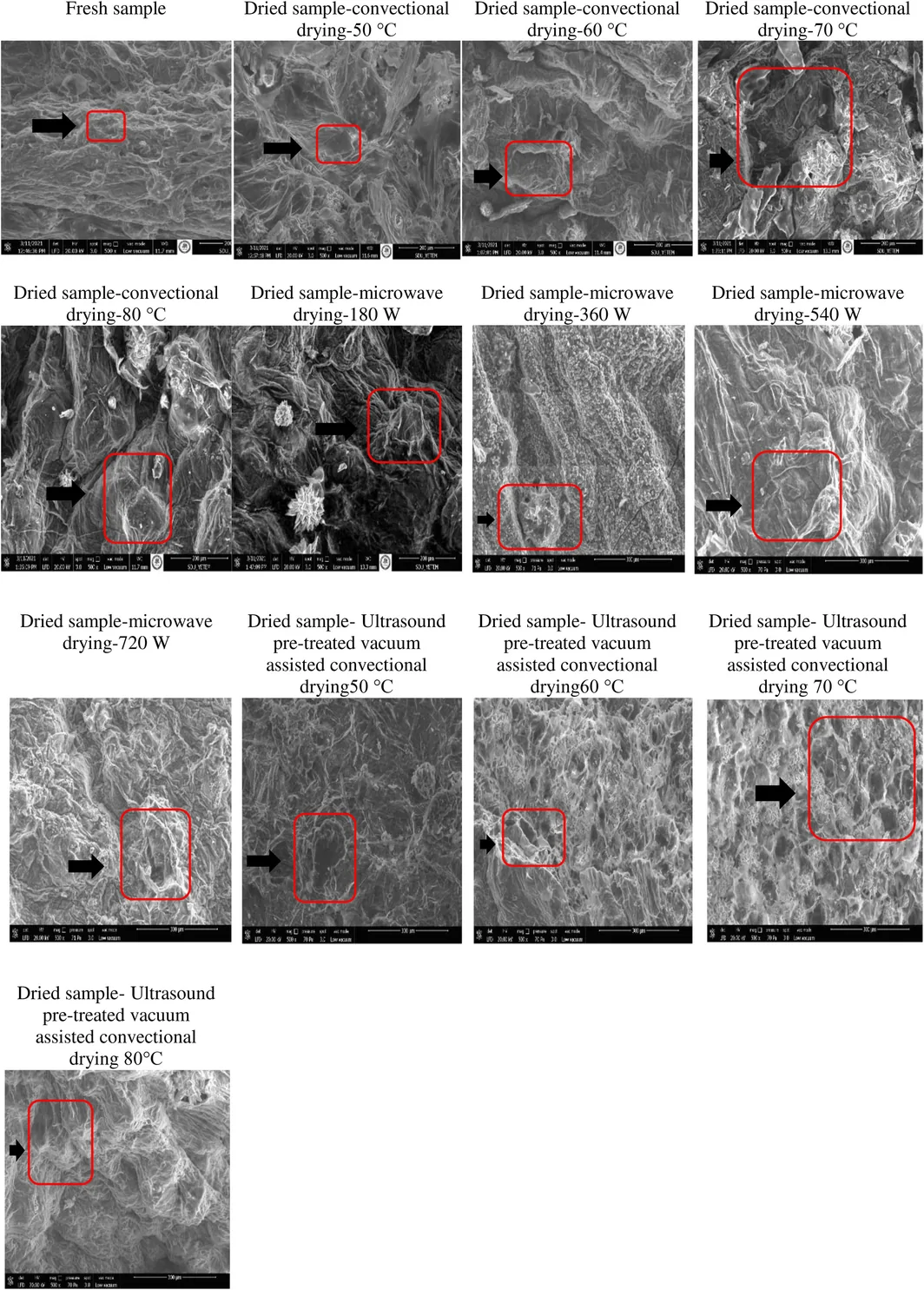
Tissue of fresh and dried samples (SEM images).

Alternative expansions and compressions produced by ultrasound waves in the product cause cellular tissue disruption. This effect is called the “sponge effect” because it resembles the appearance of a sponge when compressed and released. Acoustic waves act on the cell repeatedly, facilitating the removal of water, thereby changing the cellular structure. In examined samples after sonication, the cells of cactus cladode tissue were distorted, numerous of them were damaged or merged together forming larger spaces (Bagheri and Dinani 2019; Nowacka and Wędzik 2016; Ortuño et al. 2010). However, while these porous structures were evident at 60°C and 70°C, they decreased at 50°C as a result of the long drying time and at 80°C with the effect of temperature.

A limitation of the present study is the small number of independent drying runs (*n* = 2). Although three analytical measurements were performed for each independent drying run, these measurements were technical replicates and therefore did not constitute independent experimental units. The statistical analyses were based on the means of the technical replicates from the two independent drying runs. Consequently, the statistical findings should be interpreted cautiously, particularly with respect to the reproducibility of the drying treatments. The results should be considered preliminary, and further studies involving a larger number of independent drying runs are warranted to confirm the present findings and provide greater statistical power.

## 4 Conclusion

Interest in *O. ficus indica* has increased in the last decade, but numerous scientific papers are concerned with the fruit of the prickly pear.

This study evaluated the effects of different drying techniques on the physicochemical properties, antioxidant activity, and phenolic profile of *O. ficus indica* cladodes. The findings provide additional information on the influence of drying methods on cladode quality and contribute to the currently limited literature on the processing of cactus cladodes. Three different drying processes decreased the amount of fresh sample phenolic compounds (*p* < 0.05). As a result of the HPLC analysis, a total of nine components were determined: eight phenolic acids (gallic acid, protocatechic acid, syringic acid, ellagic acid, m‐coumaric acid, caffeic acid, p‐coumaric acid, ferulic acid) and one flavonoid (chrysin).

Ultrasound pretreatment was found to be important for total phenolic compound preservation. Among the dried samples, the highest total phenolic content was observed in ultrasound‐pretreated vacuum‐assisted convectional drying samples at 70°C. The effect of the ultrasound pretreatment was observed in the ∆*E* values and texture values of the product, and in the SEM analysis, it was determined that the cavitation effect that occurred during the ultrasound process created new cells in the structure of the 60°C and 70°C samples. Significant decreases were observed in the amounts of flavonoids and vitamin C in this drying method with the ultrasound pretreatment done in pure water.

Since the drying times of microwave and convectional drying processes are shorter and there is no difference in antioxidant capacity values between all drying techniques, the use of these two drying techniques may be an alternative to the industry.

## Author Contributions

**Şehriban Gül Bağirsakci:** data curation, resources, writing – review and editing, writing – original draft, funding acquisition, investigation, formal analysis, software, visualization, project administration. **Erkan Karacabey:** writing – review and editing, funding acquisition, methodology, supervision, data curation, project administration, conceptualization, validation, investigation, resources. **Salih Karasu:** project administration, resources, supervision, data curation, methodology, conceptualization, funding acquisition, investigation, writing – review and editing, validation.

## Funding

This work was supported by Süleyman Demirel Üniversitesi (FDK‐2019‐7364) and Yildiz Technical University.

## Ethics Statement

This study does not involve any human or animal testing.

## Conflicts of Interest

The authors declare no conflicts of interest.

## Data Availability

The data that support the findings of this study are available from the corresponding author upon reasonable request.
